# Kartagener syndrome with partial anomalous pulmonary venous drainage: a case report

**DOI:** 10.1186/s13256-026-06191-9

**Published:** 2026-06-30

**Authors:** Dwika Rasyid Firmanda, Alisia Yuana Putri, Cornelia Ghea, Asiyah Nurul Fadila, Emil Prabowo, Vammy Valentine, Fauzan Hamadah

**Affiliations:** 1 Department of Cardiology and Vascular Medicine, Dr Soetomo General Academic Hospital, Surabaya, East Java Indonesia; 2 Department of Cardiology and Vascular Medicine, Faculty of Medicine, Universitas Airlangga, Surabaya, East Java Indonesia

**Keywords:** Kartagener syndrome, Congenital anomalies, Partial anomalous pulmonary vein drainage, Bronchiectasis, Case report

## Abstract

**Background:**

Kartagener syndrome, defined by situs inversus, bronchiectasis, and chronic sinusitis. This condition rarely coexists with cardiac anomalies.

**Case presentation:**

We report the case of a 34-year-old Asian female of East Javanese descent presenting with an acute exacerbation of respiratory symptoms, including progressive dyspnea, fever, and productive cough. Clinical evaluation revealed severe hypoxemia and previously undiagnosed dextrocardia, necessitating immediate stabilization and further diagnostic workup. Imaging revealed situs inversus totalis with partial anomalous pulmonary venous drainage, where two right pulmonary veins drained into the right atrium, accompanied by severe tricuspid and moderate pulmonary regurgitation. High-resolution computed tomography (CT) confirmed bilateral bronchiectasis and thoracoabdominal situs inversus, all findings consistent with Kartagener’s syndrome. The patient improved with pulmonary vasodilators, phosphodiesterase-5 inhibitor, and antibiotics, and was discharged in stable condition.

**Conclusion:**

This case highlights the rare coexistence of Kartagener syndrome and partial anomalous pulmonary venous drainage, underscoring the crucial role of multimodality imaging in early recognition and tailored management.

## Background

Kartagener syndrome (KS) is a rare inherited condition that follows an autosomal recessive pattern. It is classically recognized by the triad of complete or incomplete situs inversus, chronic sinusitis, and bronchiectasis [1, 2]. The underlying cause is primary ciliary dyskinesia (PCD), a defect in ciliary motion that disrupts mucociliary clearance and predisposes patients to recurrent respiratory infections. Its estimated incidence ranges between 1 in 10,000 and 40,000 individuals [3, 4]. Clinically, KS often presents with symptoms of persistent cough, wheezing, nasal congestion, and repeated episodes of pneumonia. Airway infections mostly occur in childhood. Due to the non-specific presentation, these symptoms are frequently mistaken for more common respiratory disorders such as asthma, chronic bronchitis, or tuberculosis, which can delay accurate diagnosis. Imaging plays a central role in recognition, with dextrocardia on chest radiographs providing an important early clue, and high-resolution computed tomography confirming the associated bronchiectasis and sinus disease [5, 6].

While Situs Inversus Totalis is the hallmark feature of KS, the presence of additional congenital cardiac malformations is unusual. One of the rarest yet clinically important associations is partial anomalous pulmonary venous drainage (PAPVD), a condition in which pulmonary veins return abnormally to the right atrium, creating right-sided volume overload, valvular dysfunction, and progressive cardiopulmonary complications [7]. The combination of KS and PAPVD highlights the value of comprehensive multimodality imaging, including echocardiography and cardiac CT in complex anatomy and guiding appropriate clinical management.

## Timeline

2024-04-08

Initial presentation with dyspnea; performed chest X-ray, complete lab works, and echocardiography revealing suspected PAPVD; HRCT was planned for further evaluation.

2024-04-10

HRCT findings confirm APVD with additional diagnosis of bronchiectasis.

2024-04-20

Positive response to therapy given during admission; transitioned to out patient care with scheduled clinical follow-up.

## Case presentation

A 34-year-old Asian female of East Javanese descent with a history of bronchiectasis—diagnosed via chest CT in 2008 and managed with budesonide/formoterol, fenoterol, and tiotropium for the past two years—presented with a one-week history of worsening dyspnea, productive cough with purulent sputum, fever, anorexia, and malaise. There was no relevant family history. She was referred to our facility for further evaluation of suspected dextrocardia.

**Fig. 1:**
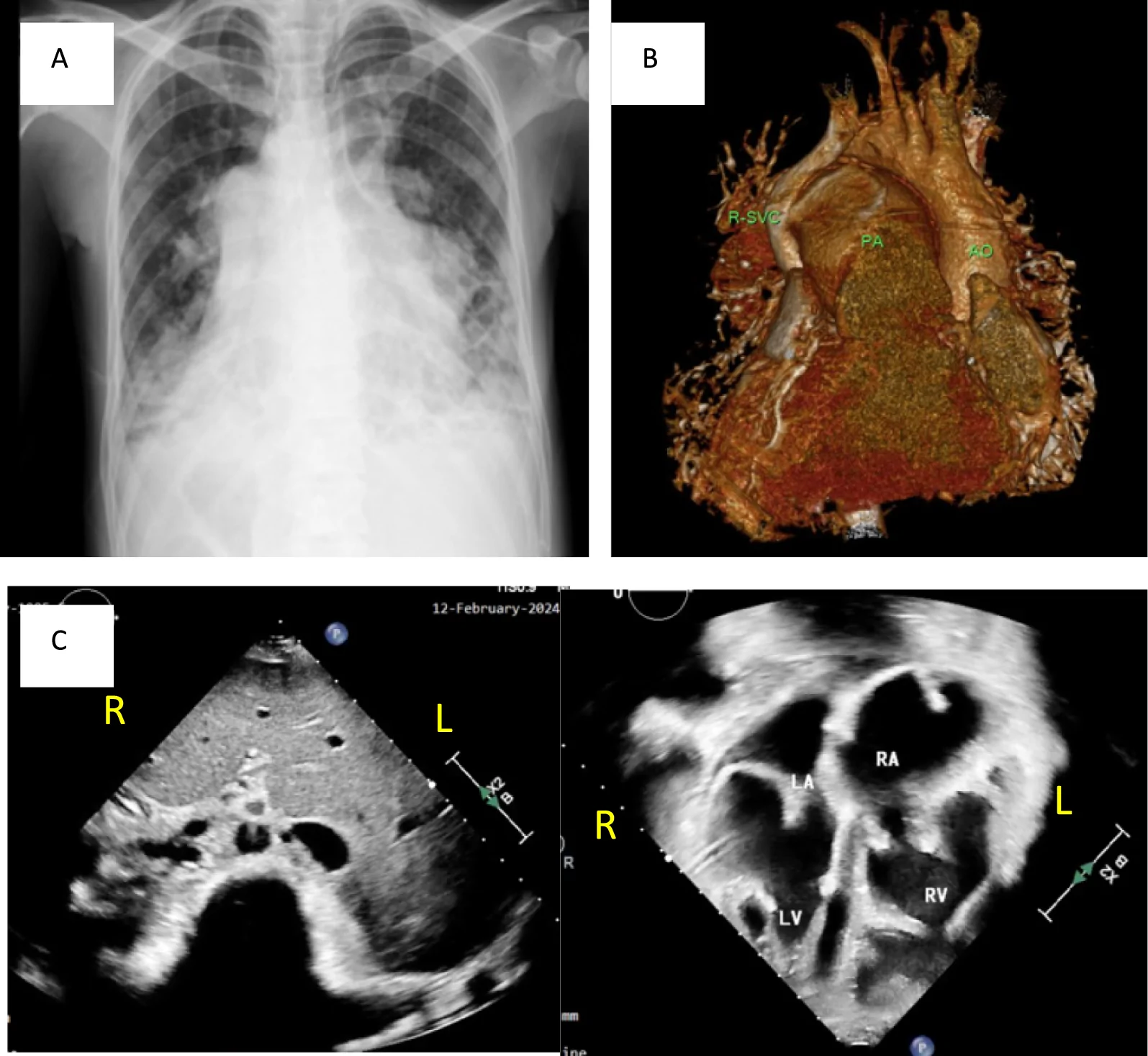
Dextrocardia, situs inversus shown in **A** chest X-ray; **B** cardiac computed tomography reconstruction; **C** transthoracic echocardiography

She appeared weak and underweight. Vital signs were within normal limit except for oxygen saturation dropped to 60% on room air which improved to 73% with a non-rebreathing mask. Auscultation revealed a grade III/VI systolic murmur at the left parasternal area, bilateral crackles, and wheezing. Chest X-ray demonstrated cardiomegaly with dextrocardia (Fig. 1).

Laboratory evaluation showed leukocytosis (WBC 14.97 × 10⁹/L). Arterial blood gas analysis revealed pH 7.41, pCO₂ 43 mmHg, pO₂ 41 mmHg, HCO₃⁻ 27.3 mmol/L, BE + 2.7, and a P/F ratio of 210, consistent with severe hypoxemia

**Fig. 2:**
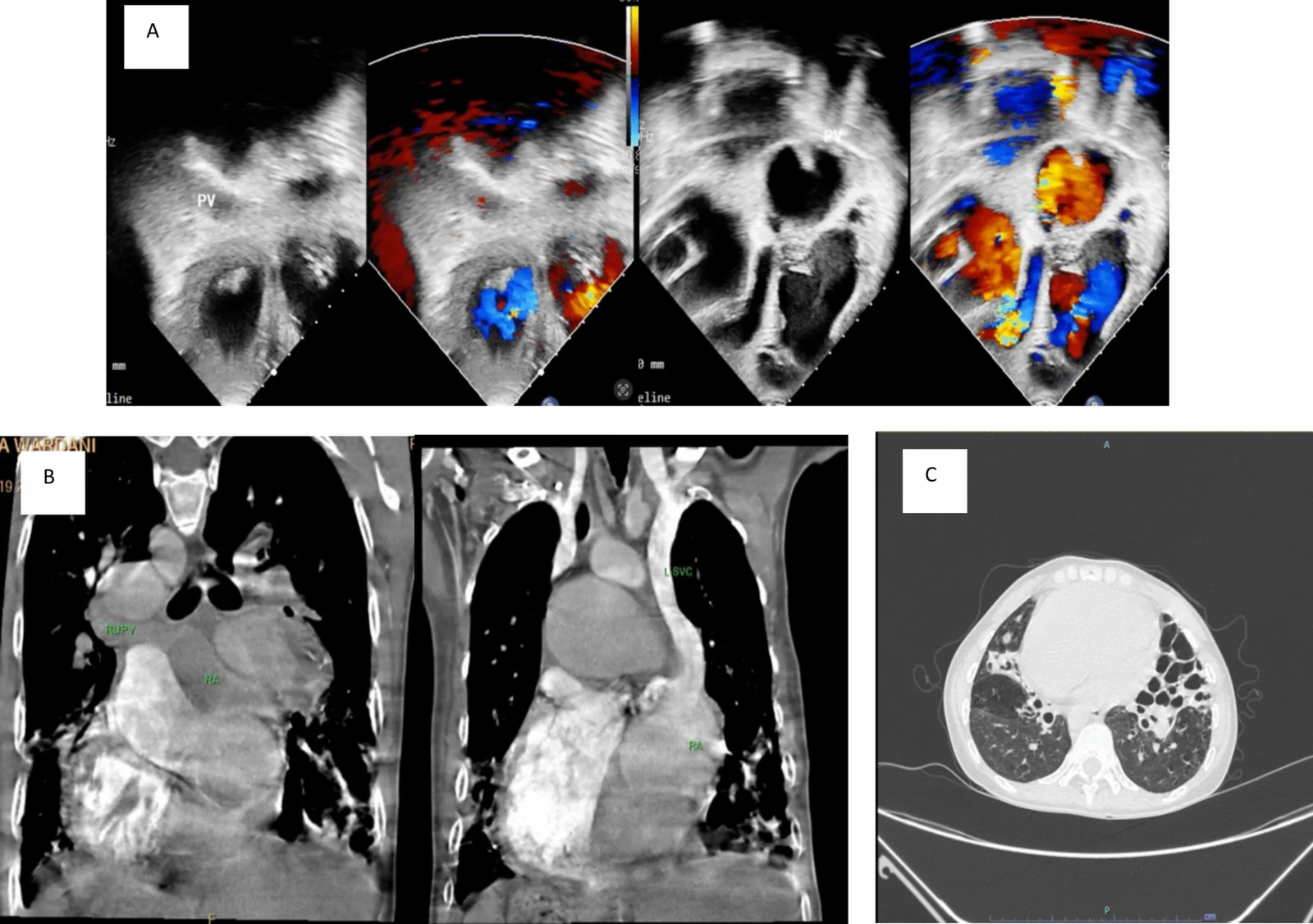
**A** Partial anomalous pulmonary venous drainage (PAPVD) shown in transthoracic echocardiography; **B** PAPVD shown in cardiac computed tomography reconstruction; **C** bronchiectasis in the middle land lower right lobe shown in high-resolution computed tomography

Echocardiography demonstrated dextrocardia, Situs Inversus Totalis, and PAPVD with severe tricuspid regurgitation and moderate pulmonary regurgitation (Fig. 2).

Cardiac CT confirmed dextrocardia, Situs Inversus Totalis, and partial anomalous pulmonary venous drainage (PAPVD) with two right pulmonary veins draining into the right atrium. Pulmonary arteries were dilated, bilateral SVC was present. High-resolution thoracic CT revealed bilateral cystic bronchiectasis and situs inversus of thoracoabdominal organs (Fig. 2).

The patient was treated with inhaled pulmonary vasodilator therapy, oral phosphodiesterase-5 inhibitor, and intravenous antibiotics, and was discharged in stable condition.

## Discussion

Kartagener syndrome (KS) is a subtype of PCD characterized by the classic triad of situs inversus, chronic sinusitis, and bronchiectasis. It is an autosomal recessive condition caused by mutations affecting motile cilia structure or function, leading to impaired mucociliary clearance. The incidence of KS is estimated to be 1 in 10.000 to 40.000 live births. The incidence of congenital heart disease in KS is analogous with that in the general population. Several studies have reported congenital heart disease in KS such as transposition of the great arteries (TGA), ventricular septal defect (VSD), pulmonary stenosis (PS), atrial septal defect (ASD) have been reported. Alas, pulmonary venous drainage anomaly in KS is not studied. [1, 2, 8–11]

Partial anomalous pulmonary venous drainage (PAPVD), is a congenital cardiovascular anomaly, in which one or more pulmonary veins drain into the right atrium or systemic venous circulation. It is considered rare whose prevalence is 0.7% based on autopsy and 0.1% based on CT scans [8]. Unlike total anomalous drainage (TAPVD), which presents in neonates, PAPVD often remains asymptomatic until later in life and may be discovered incidentally or when a patient is evaluated for unexplained right heart dilation or pulmonary hypertension. [7, 12]

The coexistence of KS and PAPVD in the same patient represents an exceedingly uncommon overlap of two congenital conditions. While KS primarily affects the respiratory tract and situs orientation, it can occasionally be accompanied by other malformations, including congenital heart defect [13]. Indeed, situs inversus in KS usually implies a structurally normal heart, but rare concurrent anomalies like PAPVD may occur, as seen in this case. Recognizing this overlap is important which can both contribute to symptoms (for example, exercise intolerance, hypoxemia) and may lead to multifactorial pulmonary arterial hypertension (PH) over time [1]. This case underscores the need for suspicion and comprehensive evaluation in patients with KS, so that coexisting cardiac anomalies such as PAPVD are not missed. This case highlights a rare presentation of Kartagener syndrome, uniquely complicated by undiagnosed PAPVD. While Situs Inversus Totalis is characteristic of Kartagener syndrome, coexisting structural heart defects like PAPVD are uncommon, yet significantly contribute to right heart volume overload and respiratory symptoms. The severe tricuspid and moderate pulmonary regurgitation observed here underscore the hemodynamic impact of this rare combination.

Early radiologic recognition was pivotal in KS especially in our case, with multiple imaging modalities delineating the full spectrum of abnormalities. Chest radiography provided the first crucial clue: it revealed dextrocardia (cardiac apex on the right) with a right-sided gastric bubble, confirming a Situs Inversus Totalis, an essential sign of Kartagener syndrome [1]. The plain X-ray also showed diffuse reticulonodular opacities consistent with bronchial wall thickening and possible bronchiectasis in the lower lung zones, prompting further investigation. High-resolution CT (HRCT) of the chest was then performed, which definitively demonstrated bronchiectasis (dilated, thick-walled bronchi) in multiple lobes, in keeping with chronic infection and impaired mucociliary clearance in PCD [14]. Note that bronchiectasis is a hallmark of Kartagener's syndrome, predominantly affecting the middle lobe, lingula, and lower lobes, as seen in our case. The HRCT also confirmed mirror-image anatomy of the thoracoabdominal organs (for example, right-sided heart and aortic arch, left-sided liver and trilobed left lung), establishing the diagnosis of Situs Inversus Totalis.

Echocardiography served as a valuable imaging to assess cardiac anatomy and function in the context of dextrocardia. It is safe, easy and readily accessible, making this modality the second line examination following plain chest radiography. The transthoracic echo helps confirmed the heart’s orientation, chamber connection, valve function, intracardiac shunt. This case shows rightward heart orientation with normal chamber connections (Situs Inversus Totalis with dextrocardia, but no major intracardiac defects such as septal defects were initially evident). However, the transthoracic echocardiography raised suspicion for an anomalous pulmonary venous return. To further evaluate the pulmonary venous system, contrast-enhanced cardiac CT angiography was obtained. The imaging confirmed a right-sided partial anomalous pulmonary venous drainage (PAPVD), with the right upper pulmonary vein emptying into the superior vena cava. This highlights the limitations of echocardiography and the enhanced sensitivity of CT angiography in characterizing anomalous pulmonary venous connections. This case underscores the superior diagnostic yield of cardiac CT over transthoracic echocardiography for detecting complex anomalous cardiovascular anatomy [6, 8, 15, 16]. No other pulmonary veins were anomalous, and no overt atrial septal defect was visualized on CT (suggesting either a small defect or a venous connection above the atrial septum).

This case highlights the integration of multimodality imaging in the establishment of comprehensive diagnosis, from the most simple and safe to the more advance and risk-carrying examination. The chest X-ray helps pointing to KS, the transthoracic echocardiography helps suggesting PAPVD, an HRCT aids the confirmation of bronchiectasis and Situs Inversus Totalis, and eventually the cardiac CT which pinpoints the anomalous venous drainage. Altogether from the examination make the diagnosis of Kartagener syndrome with PAPVD.

Currently there is no definitive cure for Kartagener syndrome, and the management is focused on controlling infections, preserving lung function, and addressing complications. A cornerstone of therapy is aggressive infection control and airway clearance. Patients are educated on daily chest physiotherapy techniques and devices to mobilize secretions from their bronchiectasis airways. Regular exercise is also encouraged to improve airway clearance. Prophylactic or suppressive antibiotic regimens may be used in patients with frequent exacerbations, and prompt culture-directed antibiotics are given for pulmonary infections [17].

For the PAPVD component, management strategy depends on the hemodynamic impact. In our patient, a single pulmonary vein was anomalously draining into the systemic venous circulation. If the shunt fraction is significant (especially if an undiagnosed associated atrial septal defect is present), surgical correction may be indicated. Cardiac surgery would involve rerouting the anomalous vein to the left atrium (sometimes with a patch closure of any atrial septal defect). We referred our patient to an adult congenital heart disease specialist to evaluate surgical options. Pulmonary vasodilator therapy was considered due to the presence of multifactorial PH. The role of pulmonary arterial hypertension (PAH)-specific medications (such as phosphodiesterase-5 inhibitors, endothelin receptor antagonists, or prostacyclin analogs) in PAPVD is not firmly established, there are case reports and small series suggesting symptomatic improvement with medical therapy, but no randomized trials exist [18–20]. Following a multidisciplinary discussion, we initiated a pulmonary vasodilator (a phosphodiesterase-5 inhibitor) to address the elevated pulmonary artery pressures, with plans to re-evaluate hemodynamic in this patient. Encouragingly, treating the patient’s chronic hypoxemia and controlling the infection may alleviate vasoconstriction in the pulmonary vasculature, potentially improving PH that is partly hypoxia-driven [21].

Comprehensive long-term follow-up is essential for a patient with both KS and PAPVD. We scheduled regular follow-up in a specialized clinic with both pulmonology and cardiology input.

## Conclusion

This case illustrates a rare coexistence of Kartagener syndrome and PAPVD. In patients with asymptomatic PAPVD, early recognition of this congenital overlap is essential for appropriate management and improved outcomes. A thorough multimodality imaging ranging from chest X-ray, TTE, to CT-scan is essential in diagnosing such case.

## Data Availability

Data sharing is not applicable to this article as no data sets were generated or analyzed during the current study.

## References

[1] Sha YW, Ding L, Li P. Management of primary ciliary dyskinesia/Kartagener′s syndrome in infertile male patients and current progress in defining the underlying genetic mechanism. Asian J Androl. 2014;16(1):101. 24369140 10.4103/1008-682X.122192 PMC3901865

[2] Ibrahim R, Daood H. Kartagener syndrome: a case report. Canadian J Respirat Therapy. 2021;21(57):44–8. 10.29390/cjrt-2020-064 PMC8059757 33912655

[3] Vanaken GJ, Bassinet L, Boon M, Mani R, Honoré I, Papon JF, *et al*. Infertility in an adult cohort with primary ciliary dyskinesia: phenotype–gene association. Eur Respir J. 2017;50(5): 1700314. 29122913 10.1183/13993003.00314-2017

[4] Mishra M, Kumar N, Jaiswal A, Verma A, Kant S. Kartagener′s syndrome: A case series. Lung India. 2012;29(4):366. 23243352 10.4103/0970-2113.102831 PMC3519024

[5] Arora A, Sirohi T, Sonalika. Kartagener’s Syndrome. Telangana J IMA. 2024;4(2):88–90.

[6] Ouali AE, Bounnite I, Moussaoui S, Labied M, Mountassir C, Lembarki G, *et al*. Clinical manifestations and diagnostic challenges of Kartagener syndrome. Int J Adv Res (Indore). 2024;12(06):909–13.

[7] Patel H, Bhutani S, Shamoon F, Virk H. Deciphering a case of pulmonary hypertension in a young female: Partial anomalous pulmonary venous drainage the culprit. Ann Thorac Med. 2018;13(1):55. 29387257 10.4103/atm.ATM_148_17 PMC5772109

[8] Pendela VS, Tan BEX, Chowdhury M, Chow M. Partial Anomalous Pulmonary Venous Return Presenting in Adults: A Case Series With Review of Literature. Cureus. 2020. 10.7759/cureus.8388 PMC7331899 32637270

[9] Tkebuchava T, von Segesser LK, Niederhäuser U, Bauersfeld U, Turina M. Cardiac Surgery for Kartagener Syndrome. Pediatr Cardiol. 1997;18(1):72–3. 8960500 10.1007/s002469900115

[10] Ida Ayu Devi A, Widyaningsih PD. Sindrom Kartagener pada perempuan dewasa: sebuah laporan kasus langka. Intisari Sains Medis. 2024;15(2):552–5.

[11] Vaid R, Fareed A, Ahmad SM. Kartagener’s Syndrome Complicated by Bronchiectasis with Tricuspid and Mitral Valve Regurgitation: A Case Report. Clin Med Insights Case Rep. 2024;3:17. 10.1177/11795476241251940 PMC11069330 38706639

[12] Mittal V, Shah A. Situs Inversus Totalis: The Association of Kartagener’s Syndrome With Diffuse Bronchiolitis and Azoospermia. Vol. 48, Arch Bronconeumol. 2012.: www.archbronconeumol.org 10.1016/j.arbres.2011.09.009 22243809

[13] Chuhwak E. Kartagener syndrome in a Nigerian African - A case report and literature review. Nigerian Journal of Medicine. 2010;18(4). 10.4314/njm.v18i4.51258 20120152

[14] Kennedy MP, Noone PG, Leigh MW, Zariwala MA, Minnix SL, Knowles MR, *et al*. High-resolution CT of patients with primary ciliary dyskinesia. AJR Am J Roentgenol. 2007;188(5):1232–8. 17449765 10.2214/AJR.06.0965

[15] Gupta S, Kasliwal R, Handa K, Bajpai P. A case of Kartagener′s syndrome: importance of early diagnosis and treatment. Indian J Hum Genet. 2012;18(2):263. 23162311 10.4103/0971-6866.100787 PMC3491309

[16] Daouda D, Carole BT, Abdoulfatihi S, Houria T, Abdellatif S, Najwa T, *et al*. Kartagener’s Syndrome a Case Report and Literature Review. SAS Journal of Medicine. 2022;8(2):117–20.

[17] McManus IC, Mitchison HM, Chung EM, Stubbings GF, Martin N. Primary ciliary dyskinesia (Siewert’s / Kartagener’s Syndrome): Respiratory symptoms and psycho-social impact. BMC Pulm Med. 2003;3(1):4. 14641928 10.1186/1471-2466-3-4 PMC317322

[18] Sears EH, Aliotta JM, Klinger JR. Partial anomalous pulmonary venous return presenting with adult-onset pulmonary hypertension. Pulm Circ. 2012;2(2):250–5. 22837866 10.4103/2045-8932.97637 PMC3401879

[19] El-Kersh K, Homsy E, Daniels CJ, Smith JS. Partial anomalous pulmonary venous return: A case series with management approach. Respir Med Case Rep. 2019;1:27. 10.1016/j.rmcr.2019.100833 PMC6456451 31008046

[20] Condliffe R, Clift P, Dimopoulos K, Tulloh RMR. Management dilemmas in pulmonary arterial hypertension associated with congenital heart disease. Vol. 8, Pulmonary Circulation. SAGE Publications Ltd; 2018. 10.1177/2045894018792501 PMC6161209 30033821

[21] Bush A, Chodhari R, Collins N, Copeland F, Hall P, Harcourt J, *et al*. Primary ciliary dyskinesia: current state of the art. Arch Dis Child. 2007;92(12):1136–40. 17634184 10.1136/adc.2006.096958 PMC2066071

